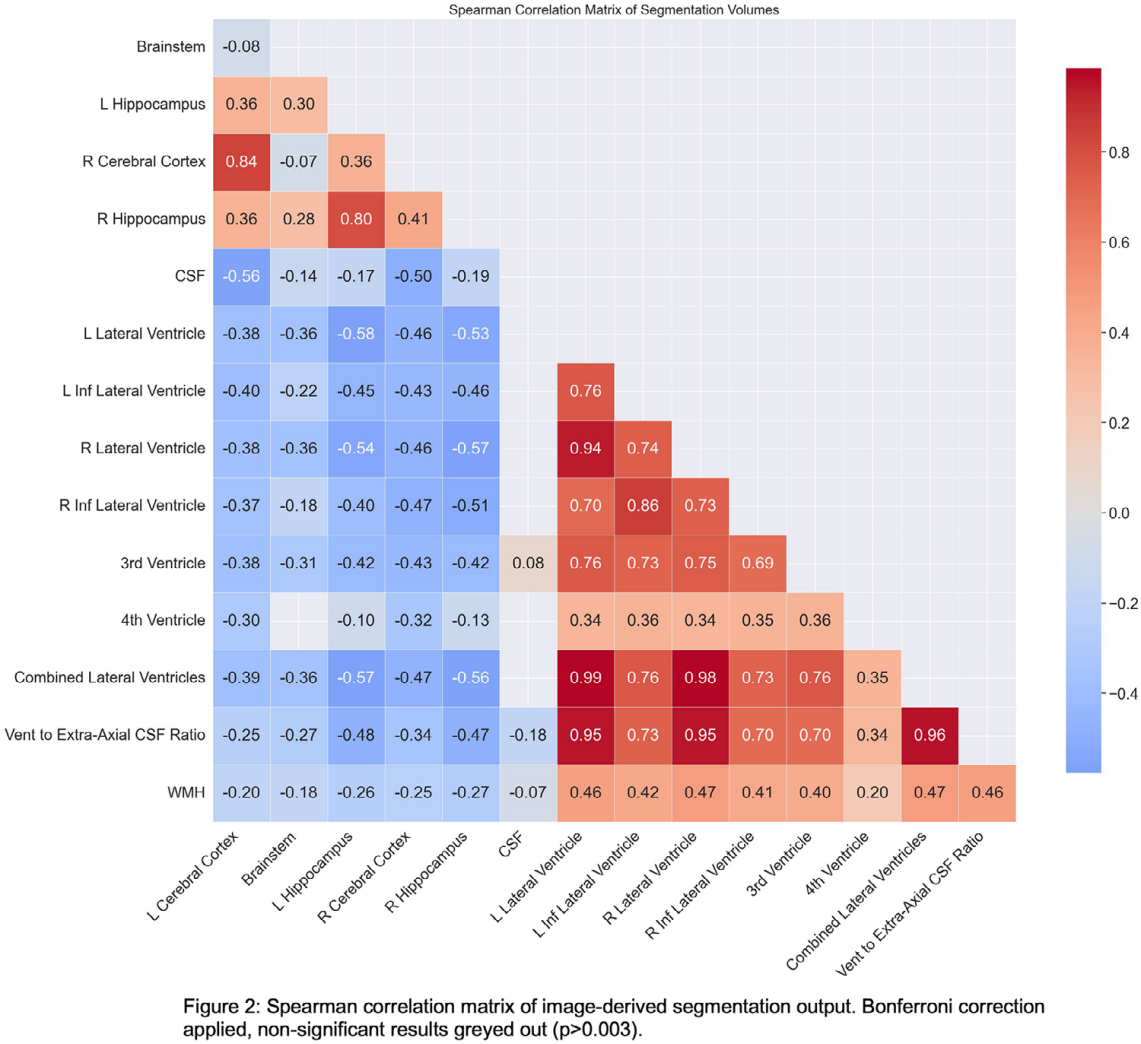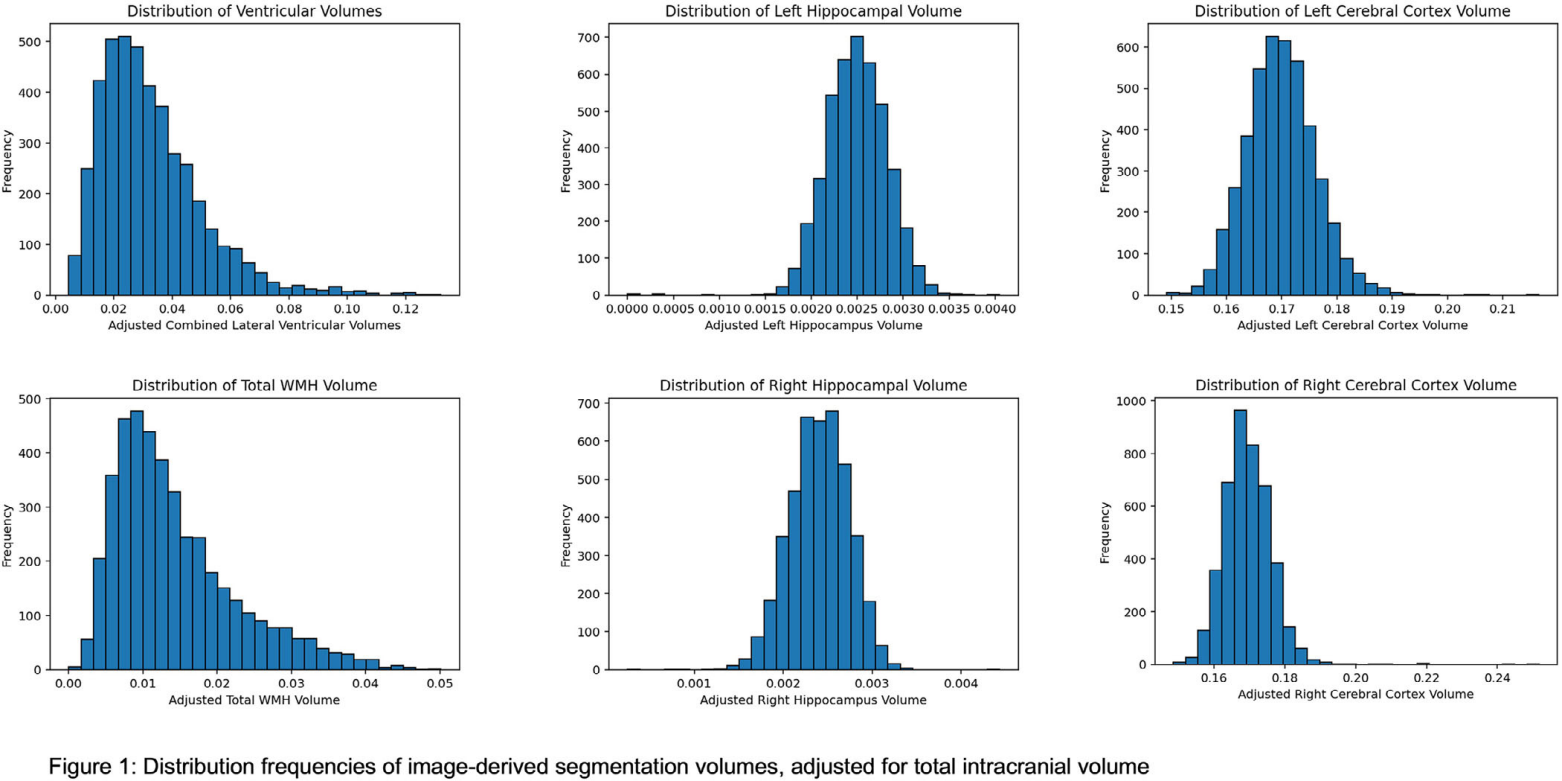# Brain imaging‐derived phenotypes in an ethnically and socio‐economically diverse memory clinic population in South London

**DOI:** 10.1002/alz70861_108002

**Published:** 2025-12-23

**Authors:** Clara Belessiotis‐Richards, Robert Leech, Robert Stewart, Gill Livingston, František Váša, Ashwin V Venkataraman

**Affiliations:** ^1^ Department of Psychological Medicine, Institute of Psychiatry, Psychology and Neuroscience, Kings' College London, London UK; ^2^ Institute of Psychiatry, Psychology and Neuroscience, King's College London, London UK; ^3^ NIHR Biomedical Research Centre for Mental Health and Biomedical Research Unit for Dementia at South London and Maudsley NHS Foundation, London UK; ^4^ Department of Psychological Medicine, King's College London, London, London UK; ^5^ Psychiatry, University College London, London, London UK; ^6^ North London NHS Foundation trust, London, London UK; ^7^ Centre for Neuroimaging Sciences, IoPPN, Kings' College London, London, London UK; ^8^ Institute of Psychiatry, Psychology and Neuroscience, King's College London, London UK; ^9^ Centre for Neuroimaging Sciences, IoPPN, King’s College London, London UK; ^10^ South London and Maudsley NHS Foundation Trust, London UK; ^11^ Centre for Healthy Brain Ageing, IoPPN, King’s College London, London, London UK

## Abstract

**Background:**

Brain imaging data for dementia research have historically been derived from highly selected groups, leading to potentially misleading norms. Here, we aimed to describe broad imaging‐derived phenotypes, including global white‐matter hyperintensities (WMH) volume, in an ethnically and socio‐economically diverse real‐world memory clinic population for the first time.

**Method:**

Our population comprised people aged 60 years plus with at least one referral to memory services and an available brain MRI in the South London and Maudsley (SLAM) NHS Trust in England. We extracted routine clinical data on age, sex, ethnicity, and socioeconomic deprivation. Routinely‐collected brain MRIs were identified through the SLAM ImageBank. Images were segmented with SynthSeg and WMH‐SynthSeg. All volumetric measures were adjusted by total intracranial volume. We examined available regions of interest of apriori relevance to dementia and to understanding ventricular dynamics in the future, in relation to WMH volume.

**Result:**

Our final sample included 4,311 MPRAGE and 4,285 FLAIR scans. Mean age was 75.6 years (standard deviation (SD) 7.8), 57% were female. A majority (67%) of the population lived in areas at or below the median for national deprivation. 59% of the population identified as White, 21% as Black, 10% as Asian, and 10% as Other. Distributions of selected volumes are shown in Figure 1. Hippocampal volumes were positively correlated with cerebral cortical volumes (rho: 0.36 to 0.41) (Figure 2), but inversely related to lateral ventricular volumes (rho: ‐0.57 to ‐0.56), and this persisted after adjustment for extra‐axial cerebrospinal fluid (CSF) (rho: ‐0.41). Total WMH volume was positively correlated with lateral ventricular volumes (rho: 0.42) but not with total CSF (rho: ‐0.12) or cortical volumes (rho: ‐0.18 to ‐0.17).

**Conclusion:**

In general we found that volume of WMH was positively correlated with intra‐axial CSF volumes but inversely related to cortical volumes. Hippocampal and cortical volumes were inversely correlated with CSF, as expected. Improving our understanding of imaging‐derived phenotypes in routine clinical populations is crucial to the generalisability and applicability of potential future treatments for dementia. These results will form the basis for developing normative models including WMH data in a large‐scale representative clinical population.